# Time-series COVID-19 policymaker analysis of the UAE, Taiwan, New Zealand, Japan and Hungary

**DOI:** 10.1016/j.dialog.2022.100081

**Published:** 2022-11-18

**Authors:** Yoshiyasu Takefuji

**Affiliations:** Faculty of Data Science, Musashino University, 3-3-3 Ariake Koto-ku, Tokyo 135-8181, Japan

**Keywords:** COVID-19 deaths, Demographic perspective, Policy analysis tool, Scorecovid, Hiscovid

## Abstract

There are two types of policy analysis: socioeconomic analysis and public policy outcome analysis. The socioeconomic analysis is used for understanding the relationship between COVID-19 incident and mortality and building effective governance. There are two types of policy outcome analysis: general policy analysis and time series policy analysis. This paper is a policy outcome analysis of COVID-19, not a policy analysis. This paper examines COVID-19 policy outcome analysis of five countries such as the UAE, Taiwan, New Zealand, Japan and Hungary. Two policy outcome analysis tools are used in this paper such as scorecovid to generate a snapshot list of sorted scores and time-series hiscovid to identify when policymakers made mistakes for correcting mistakes in the near future policy update. Scores in both tools are based on the population mortality rate: dividing the number of COVID-19 deaths by the population in millions. The lower the score, the better the policy. The higher the score, the more deaths that make people unhappy. COVID-19 death is the most unfortunate event in life and is caused by policy. The introduced time-series policy analysis tool, hiscovid discovered ten facts of five countries. Discovered ten facts will be detailed in this paper. Visualization of policy outcomes over time will play an important role in mitigating the COVID-19 pandemic.

## 1 Introduction

Policymakers need to consider two indicators such as the socioeconomic effects of COVID-19 [1,2] and public policy outcomes. The socioeconomic status is the social standing of individuals and communities. The socioeconomic analysis is important for understanding the relationship between COVID-19 incident and mortality and building effective governance [2], but it cannot directly solve the COVID-19 pandemic for mitigating COVID-19. However, COVID-19 policy outcome analysis plays a key role in mitigating the pandemic. In other words, policy outcome analysis can reveal and identify when policymakers made mistakes in the COVID-19 pandemic. The policy causes of COVID-19 that are identified can be corrected by updating policies.

There are two types of policy outcome analysis: general policy analysis and time series policy analysis. General policy analysis is a snapshot of events, whereas a time-series policy analysis allows us to observe the progress and evolution of COVID-19 and to identify when they made mistakes over time. The identified mistakes can be corrected by updating policies for preventing the similar mistakes in the future.

Introduced scorecovid is a snapshot policy outcome analysis tool while hiscovid is a time-series policy outcome analysis tool. To our knowledge, there is no COVID-19 policy outcome analysis tool. Therefore, this paper focuses on analysis of the snapshot of 16 countries with the scorecovid tool and time-series COVID-19 policy outcomes of five countries with the hiscovid tool.

The proposed COVID-19 policy outcome analysis tool, hiscovid was introduced for urban governance for policymakers to identify when they made mistakes in COVID-19 policies [3,4]. The scores for individual policies in the snapshot and time series are based on the population mortality rate: the number of deaths in COVID-19 divided by the population in millions. The lower the score, the better the COVID-19. The higher the score, the more deaths that make people unhappy.

The hiscovid tool can identify when policymakers made mistakes in time-series scores. The time-series scoring is an important feature of the introduced policy outcome analysis tool. The outcome of a COVID-19 policy can be expressed by a graph. If the generated graph is a flat line, it indicates that the COVID-19 policy is indeed successful in mitigating the pandemic. If the graph is diagonal or not flat, it indicates that the COVID-19 policy is not working well. The steeper the slope of the diagonal graph, the worse the COVID-19 policy. The looser the slope of the diagonal graph, the less bad the COVID-19 policy is.

The hiscovid tool is a Python Package Index (PyPI) application which allows it to run on Windows, MacOS, and Linux operating systems as long as Python is installed on the system. According to PePy which is a site on PyPI download statistics [5], hiscovid has been downloaded by 1159 times worldwide as of October 8, 2022. The number of large downloads indicates that the applicability, the usability and the usefulness were justified.

In this paper with the hiscovid tool, the result of policy analyses of five countries such as the UAE, Taiwan, New Zealand. Japan and Hungary respectively will be discussed for mitigating the COVID-19 pandemic.

The more the deaths the higher the score. The less the deaths, the lower the score. The lower the score, the better the policy. The higher the score, the more deaths that make people unhappier. In other words, the score is a happiness index of COVID-19.

## 2 Methods and results

Data is scraped over the Internet. In scorecovid, the latest data on population is scraped from the following site: https://www.worldometers.info/world-population/population-by-country/.

In scorecovid, the latest data on total deaths is scraped from the following site: https://github.com/owid/covid-19-data/raw/master/public/data/jhu/total_deaths.csv.

In scorecovid, scoring individual country policies are calculated by dividing the total deaths by the population in millions.

In hiscovid, the number of daily deaths is scraped from the following site: https://covid.ourworldindata.org/data/owid-covid-data.csv.

Calculation of time-series scoring is the similar to that of scorecovid.

Two policy outcome analysis tools such as snapshot policy outcome analysis tool with scorecovid and time-series policy outcome analysis tool with hiscovid will be used for analysing COVID-19 policies of five countries such as the UAE, Japan, New Zealand, Taiwan and Hungary respectively.

scorecovid generates a snapshot list of sorted scores of countries where scoring individual policies is based on a single metric: dividing the number of COVID-19 deaths by the population in millions [6]. The lower the score, the better the policy. In other words, the outcomes of individual policies can be evaluated in terms of deaths from a demographic perspective. In the words, the score is normalized by dividing the number of deaths by the population in millions. However, the result of the scorecovid calculation is a snapshot. scorecovid cannot identify when policymakers made mistakes, whereas hiscovid can identify when policymakers made mistakes. In this paper, we use these two tools to analyse COVID-19 policies in snapshot with the scorecovid tool and in time series with the hiscovid tool.

The scorecovid tool is a PyPI application so that it can be installed by the following command as long as Python package is installed on the system. The character ($) indicates the prompt from the system terminal.

$ pip install scorecovid

Similarly, the time-series hiscovid tool can be installed by the following command.

$ pip install hiscovid

## 3 Results

In order to run scorecovid, simply run the following command.

$ scorecovid

Scorecovid will automatically download the necessary file over the Internet and show the sorted scores with the better policy in order. Modify the countries file by adding the text of United Arab Emirates. Then, run it again. Table 1 shows the result of scorecovid as of October 8, 2022.

**Table 1:** Scorecovid result of 16 countries as of October 8, 2022: score is calculated by the number of deaths divided by the population in millions.

Country	Deaths	Population	Score
United Arab Emirates	2346	9.89	237.2
Japan	45533	126.48	360
New Zealand	2038	4.82	422.8
Taiwan	11465	23.82	481.3
South Korea	28675	51.27	559.3
Australia	15369	25.5	602.7
Iceland	213	0.34	626.5
Canada	45646	37.74	1209.5
Israel	11710	8.66	1352.2
Germany	150535	83.78	1796.8
Sweden	20274	10.1	2007.3
France	155491	65.27	2382.3
United Kingdom	207528	67.89	3056.8
United States	1062560	331	3210.2
Brazil	686706	212.56	3230.6
Hungary	47576	9.66	4925.1

Countries with the mandatory or voluntary test-isolation policy include the UAE, Japan, New Zealand, Taiwan, South Korea, Australia and Iceland while countries with no test-isolation policy include Canada, Israel, Germany, Sweden, France, the UK, the US, Brazil and Hungary. The test-isolation policy is to test and identify infected individuals at an early stage and to isolate them from uninfected people during the quarantine period.

hiscovid is a time-series COVID-19 policy outcome analysis tool to identify when policymakers made mistakes and the result can be used for correcting their mistakes in the near future policy update. After installing hiscovid, run the following command for generating results of five countries such as the UAE, Taiwan, New Zealand. Japan and Hungary. The lower the score, the better the COVID-19 policy. The vertical axis indicates time-series scores in generated graphs.

Five countries such as the UAE, Taiwan, New Zealand, Japan and Hungary can be analysed by the following two commands. For ease of understanding in policy outcome analysis, hiscovid was used twice.

$ hiscovid ‘United Arab Emirates’ Taiwan ‘New Zealand’ Japan

$ hiscovid Hungary ‘New Zealand’

**Fig. 1:**
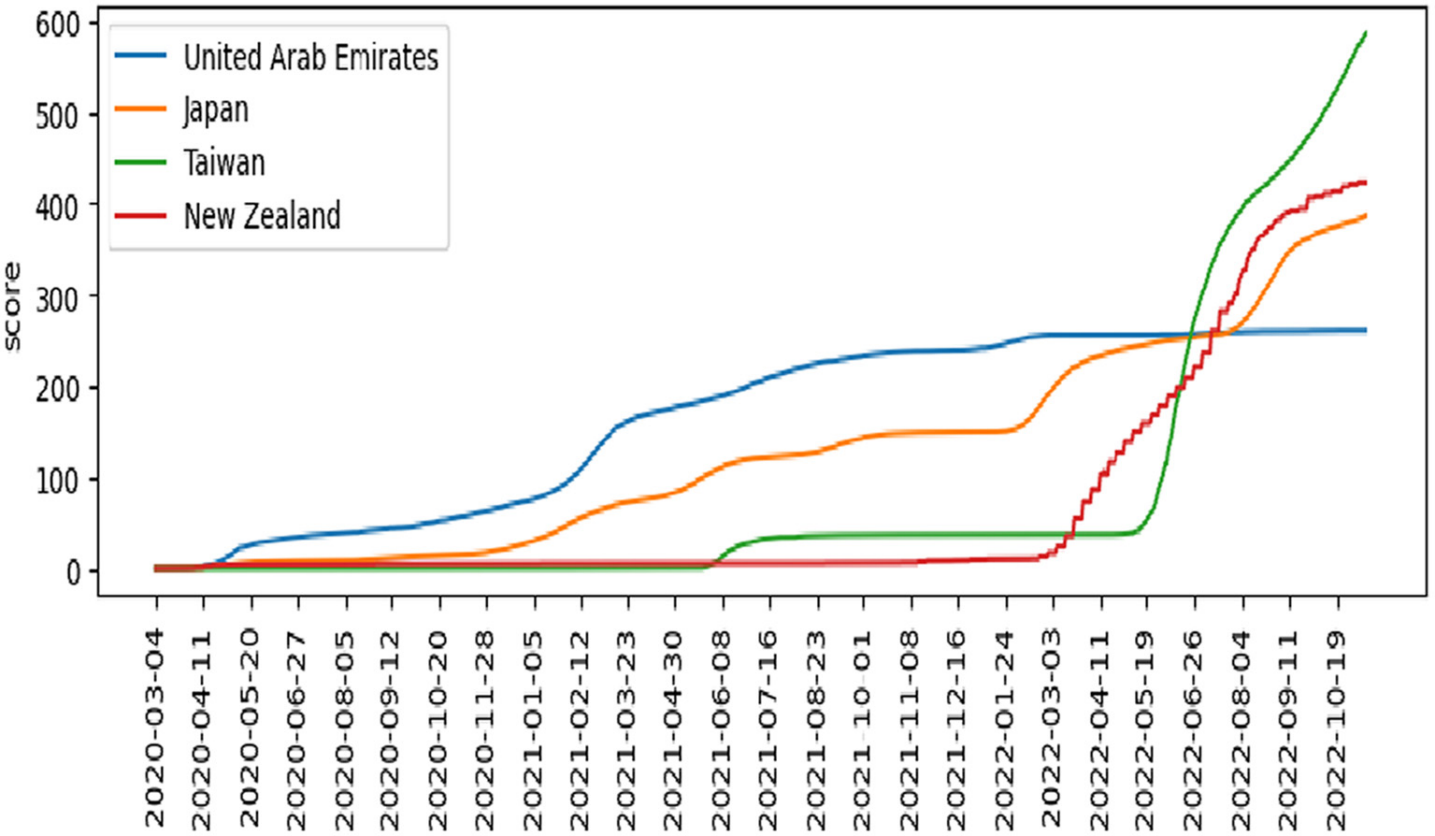
Hiscovid result of the UAE, Japan, Taiwan and New Zealand as of Nov. 13, 2022.

**Fig. 2:**
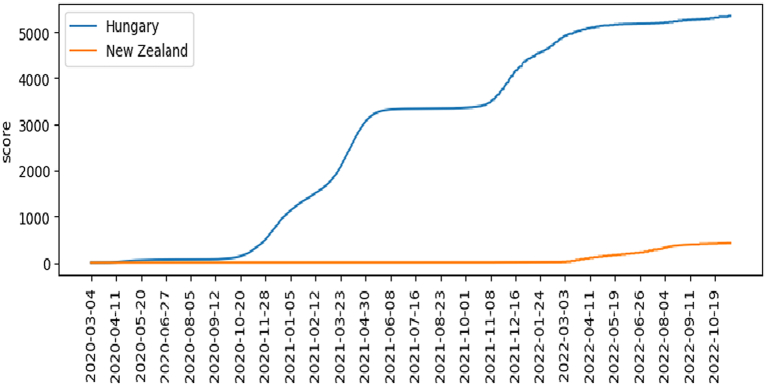
hiscovid result of New Zealand and Hungary as of Nov. 13, 2022.

Four graphs for the UAE, Taiwan, New Zealand and Japan were generated by the above command as shown in Fig. 1 as of Nov. 13, 2022. Fig. 2 compares the results for New Zealand and Hungary. The horizontal axis is the date of the country score and the vertical axis is the individual score for each country.

## 4 Discussions

In Table 1 generated by scorecovid, the UAE, Japan, New Zealand, and Taiwan have the highest scores, in that order. Hungary has the worst score as of October 8, 2022. The scorecovid tool is intended to be used for poorly scored countries to learn good strategy from countries with excellent scores. The score 237.2 of the UAE is 20 times better than that 4925.1 of Hungary.

Fig. 1 discovers the following nine facts:

1. Taiwan made two mistakes in May 2021 and May 2022.
2. There is a flat graph in Taiwan except May-June 2021 until May 2022
3. New Zealand made a single mistake in March 2022.
4. There is a flat graph in New Zealand until March 2022.
5. There is no flat graph in Japan.
6. Japan made many small mistakes.
7. From January 2022, there is a flat graph in the UAE.
8. The strong resurgence was observed in the UAE in January 2022
9. While the COVID-19 epidemic is now well controlled in the UAE, Japan, Taiwan, and New Zealand have recently seen a strong resurgence of COVID-19.
  Fig. 2 reveals that Hungary made two big mistakes around October 2020 and October 2021 respectively. The hiscovid tool discovered the total of ten facts of five countries.

COVID-19 vaccination in the UAE began in January 2021 [7,8]. According to the COVID-19 vaccine tracker, the UAE is with 99.01% fully vaccinated and 52.0% boosting. The UAE started providing a booster shot against COVID-19 in August 2021 [8]. As of August 27, 2022, the total number of accumulated COVID-19 deaths is 2341. However, the number of daily new cases is more than 500 as of today. In other words, the trend toward the end of the COVID-19 pandemic is still not visible.

The hiscovid tool not only shows the time series of scores, but also identifies when policymakers made mistakes in the time series scores.

Fig. 1 revealed nine facts with the hiscovid tool. Fig. 2 discovered one fact of Hungary. New Zealand made a single mistake with lifting border regulations in March 2022 [9,10]. Taiwan made two mistakes such as the first mistake in May 2021 with missing test crew and their families which spread COVID-19 [11] and the second mistake in May 2022 with shortening the quarantine period to 3 days [12].

The test-isolation strategy is used in four countries such as the UAE, Taiwan, New Zealand, Japan and Hungary. The test-isolation policy is to test and identify infected individuals at an early stage and to isolate them from uninfected people during the quarantine period.

In Japan’s graph, there is no flat graph observed while the UAE’s graph is flat from February 2022. However, the results differ greatly because of the significant difference in the use of mandatory test-isolation strategy by law adopted in the UAE, Taiwan, and New Zealand, while voluntary one is used in Japan. In other words, the mandatory policy is very effective against COVID-19 by suppressing the pandemic while the voluntary one is very leaky with non-flat graph observed.

The UAE changed its quarantine policy from 14 days to 10 days in January 2021, and a strong resurgence was observed [13].

In order to suppress the COVID-19 pandemic in the UAE, the longer quarantine period should be adopted.

The snapshot and Time-series policy outcome analysis tools should be used for revealing the best policy for the future pandemic. The poorly scored countries should learn good strategies or policies from countries with excellent scores. Daily-based policy outcome analysis should be conducted to identify policy mistakes. The population mortality rate is a key indicator for calculating the snapshot and time-series scores of individual policies. The higher the score, the more deaths that make people unhappier. The lower the score the better the policy.

17 references on COVID-19 management and control suggested by reviewers are briefly summarized. Coccia presented that reproduction number does not provide preventive information to cope with future epidemics or pandemics [14]. The introduced index c contageous quantifier should be used [14]. Huang et al. studied epidemic prevention system for shared car [15]. Coccia proposed strategies of prevention of pandemic threats with the use of the index c contageous quantifier [16]. Mohamadian et al. investigated stakeholders analysis of COVID-19 management and control in Iran [17]. Núñez-Delgado et al. wrote editorial on SARS-CoV-2 and other pathogenic microorganisms in the environment [18]. Benati et al. analyzed the relationship between public governance and COVID-19 vaccinations during early 2021 [19]. Inequalities in vaccination made significant differences in Barcelona [20]. The rapid availability of geolocalized data and by socioeconomic level helped public authorities to implement targeted policies and collaborative interventions for the most vulnerable populations [20]. Without appropriate therapies and drugs in Italy, timely and widespread testing to detect and isolate all infected people reduced total deaths and negative effects of COVID-19 on people's health [21]. Laage-Thomsen et al. used the results of an interdisciplinary expert survey completed in 2020 to analyse expert perceptions [22]. Coccia showed comparative critical decisions in risk management for problem solving [23]. Marí-Dell'Olmo et al. studied inequalities and their result indicated the existence of inequalities in the incidence of COVID-19 in an urban area of Southern Europe [24]. Coccia reported pandemic prevention: lessons from COVID-19 [25]. Abrams et al. investigated social determinants of health including poverty, physical environment (eg, smoke exposure, homelessness), and race or ethnicity can have a considerable effect on COVID-19 outcomes [26]. Coccia suggested that the design of effective health policies for prevention and preparedness for future pandemics should be backed by good governance and adoption of new technologies in the country [27]. Vásquez-Vera et al. studied inequities in the distribution of COVID-19 in order to uncover and understanding these inequities to develop proper intersectoral policies to tackle this crisis [28]. Coccia investigated how to improve preparedness by developing an empirical analysis based on global data to estimate the max share of people vaccinable in relation to socioeconomic wellbeing of nations [29]. Hyland-Wood et al. suggested that an effective communication strategy is a two-way process in which clear messages are tailored to diverse audiences through appropriate platforms and shared by trusted people [30].

Wang et al. surveyed the general public in China to better understand their levels of psychological impact, anxiety, depression, and stress during the initial stage of the COVID-19 outbreak [31]. During the initial phase of the COVID-19 outbreak in China, more than half of the respondents rated the psychological impact as moderate-to-severe, and about one-third reported moderate-to-severe anxiety [31]. In other words, the psychological impact needs to be mitigated or reduced through counseling. The same group investigated a longitudinal study on the mental health of general population during the COVID-19 epidemic in China [32]. It concluded that the government should focus on effective dissemination of impartial knowledge about COVID-19, teaching correct containment methods, ensuring necessary services and supplies, and providing adequate financial support. In other words, the financial support is immediately needed for mitigating psychological anxiety. Hao et al. investigated attitudes toward COVID-19 vaccination in people suffering from depression or anxiety disorder and people without mental disorders, and their willingness to pay for it [33]. Psychiatric patients showed high acceptance and willingness to pay for the COVID-19 vaccine [33]. Tan et al. conducted an online survey on return to work during COVID-19 pandemic stress [34]. The return to work was not causing high levels of psychiatric symptoms in the workforce. This result may be useful for other countries. Lau et al. suggested that individuals with higher levels of burnout may also have higher levels of fear of COVID or vice versa [35]. The result may be as expected.

## 5 Conclusion

The proposed hiscovid tool discovered ten facts. Policymakers in the world should use the hiscovid tool to correct their mistakes for updating their policies in the near future. The hiscovid tool allows policymakers to identify their mistakes for controlling the COVID-19 pandemic. The hiscovid tool identified two important facts on the mandatory test-isolation policy and the quarantine period. the mandatory test-isolation policy with the reasonable quarantine period is very effective in suppressing the COVID-19 pandemic, the quarantine period should be carefully monitored and controlled.

The longer the quarantine period, the better the COVID-19 policy. In other words, the longer the quarantine period, the less COVID-19 spreads. The shorter the quarantined period, the more COVID-19 spreads. Visualization of time-series policy outcomes of COVID-19 can play an important role in mitigating the COVID-19 pandemic. The more the COVID-19 deaths, the unhappier we are.

There are many countries that did nothing until vaccination began. The mandatory test-isolation policy was very effective to suppress the COVID-19 pandemic. Initially, vaccination was effective, but new species with spike mutations and immune escape made vaccination less effective. Regardless of vaccination, the mandatory test-isolation was the most effective method adopted in New Zealand and Taiwan to successfully suppress the COVID-19 pandemic.

## Funding

This research has no fund.

## Data availability statement

Data is available at the following site: https://covid.ourworldindata.org/data/owid-covid-data.csv.

## Authors' contributions

YT completed this research, wrote the program and the manuscript.

## Ethics approval

Not Applicable.

## Consent to participate

Not Applicable.

## Consent for publication

Not Applicable.

## Declaration of Competing Interest

The author has no conflict of interest.
